# Novel Synthetic Opioids (NSO) Use in Opioid Dependents Entering Detoxification Treatment

**DOI:** 10.3389/fpsyt.2022.868346

**Published:** 2022-06-01

**Authors:** Michael Specka, Thomas Kuhlmann, Udo Bonnet, Jürgen Sawazki, Luzia Schaaf, Stefan Kühnhold, Renate Steinert, Torsten Grigoleit, Helmut Eich, Benita Zeiske, Antje Niedersteberg, Katharina Steiner, Fabrizio Schifano, Norbert Scherbaum

**Affiliations:** ^1^Department of Addictive Behaviour and Addiction Medicine, LVR Hospital Essen, Medical Faculty, University Duisburg-Essen, Essen, Germany; ^2^Psychosomatic Hospital Bergisch Gladbach, Bergisch Gladbach, Germany; ^3^Castrop-Rauxel Evangelic Hospital, Castrop-Rauxel, Germany; ^4^Landschaftsverband Rheinland (LVR) Hospital Viersen, Viersen, Germany; ^5^Landschaftsverband Westfalen-Lippe (LWL) Hospital Lippstadt and Warstein, Warstein, Germany; ^6^Landschaftsverband Westfalen-Lippe (LWL) Hospital Münster, Münster, Germany; ^7^Landschaftsverband Rheinland (LVR) Hospital Langenfeld, Langenfeld, Germany; ^8^Alexianer Hospital, Krefeld, Germany; ^9^Alexius/Josef Hospital, Neuss, Germany; ^10^Landschaftsverband Rheinland (LVR) Hospital Düren, Düren, Germany; ^11^Psychopharmacology, Drug Misuse, and Novel Psychoactive Substances Research Unit, School of Life and Medical Sciences, University of Hertfordshire, Hatfield, United Kingdom

**Keywords:** new synthetic opioids, fentanyl, fentanyl analogs, inpatient detoxification, opioids, opioid dependence

## Abstract

**Introduction:**

Over the last decade, the use of New/Novel Synthetic Opioids (NSO) has emerged as an increasing problem, and especially so in the USA. However, only little is known about the prevalence and history of NSO use in European heroin dependents.

**Method:**

A cross-sectional multicenter study, carried out with the means of both standardized interviews and urine toxicology enhanced screening, in a sample of opioid addicted patients referred for an in-patient detoxification treatment.

**Results:**

Sample size included here *n* = 256 patients; prior to admission, 63.7% were prescribed with an opioid maintenance treatment. Lifetime use of heroin and opioid analgesics was reported by 99.2 and 30.4%, respectively. Lifetime NSO/fentanyl use was reported by 8.7% (*n* = 22); a regular use was reported by 1.6% (*n* = 4), and ingestion over the 30 days prior to admission by 0.8% (*n* = 2). Most typically, patients had started with a regular consumption of heroin, followed by maintenance opioids; opioid analgesics; and by NSO. Self-reported data were corroborated by the toxicology screenings carried out; no evidence was here identified for the presence of heroin being contaminated by fentanyl/derivatives.

**Discussion:**

NSO and also opioid analgesics did not play a relevant role in the development and the course of opioid/opioid use disorders in German patients referred for an inpatient detoxification treatment.

## Introduction

Over the last 20 years or so, a dramatic increase in the number of deaths relating to the use of psychotropic substances, mostly from opioids, has been reported from the United States of America. The first wave mostly related to prescription opioid analgesics; the second, from 2010 and onwards, to heroin; and the current one, which started in 2014, has been associated with the intake of highly potent new/novel synthetic opioids [NSO; (1)]. From 2013 to 2019 a ten-fold increase in the age-adjusted synthetic opioid death rate was observed in the USA, accounting for approximately one half of the total of 70,630 drug overdose deaths in 2019 (2). Over the past decade, an opioid crisis has also been recorded in Canada, driven by both illicit and prescription opioid misuse (1); Australia; and in the United Kingdom (3, 4).

One could wonder if the opioid epidemic has affected a range of other countries also (5), and whether or not this may have been driven by NSO intake. NSOs include fentanyl and its analogs (e.g., acetylfentanyl, butyrfentanyl, furanylfentanyl, ocfentanil, acrylfentanyl and para-fluoroisobutyrfentanyl); other synthetic opioids such as AH-7921 (e.g., a benzoamide), U-47700 (e.g., a compound closely related to AH-7921), MT-45 (e.g., a piperazine); and many others (6–8). According to the European Monitoring Centre for Drugs and Drug Abuse (9), some 700 deaths involving fentanyl, and nearly 400 deaths involving fentanyl analogues, were reported in 2016–2017 within the European Community. Finland accounted for most cases, but 13 further countries were involved, including Estonia, Germany, Sweden, and the United Kingdom.

By far, the most dangerous among these NSOs are fentanyl and its derivatives. Compared with other opioids, they have a lower price and higher potency, fentanyl 50–100 times, and carfentanil 10,000 times, more potent than heroin and morphine, and the risk of accidental overdose is much increased (8). A possible complication of NSO use is the induction of muscle rigidity, including chest wall rigidity (“wooden chest syndrome”), which makes assisted ventilation and breathing difficult (10). In addition, higher doses or repeated dosing of the opioid antagonist naloxone are required to reverse overdoses in emergency situations, which raises concerns about the adequacy of this treatment (11).

In the USA, nearly 80 percent reported of having used prescription opioids prior to heroin (12, 13). In such cases, one could tentatively argue that a long-term prescription of opioid analgesics would be associated with both tolerance and dependence, and this would be followed by a switch to illicit opioids/opioids such as heroin and/or NSO. Concerns about growing problem of illicitly manufactured fentanyl and the possible advent of a synthetic opioid crisis also in Europe have been raised early (14).

In Germany NSOs have been detected in single cases of patients presenting with opioid overdose in emergency departments, and of fatalities (15), but as with most of the rest of Europe, NSO use still seems to be a marginal phenomenon (1). It cannot be ruled out that in part this is due to underreporting. The prevalence of NSO use is difficult to estimate; users may often be unaware of the index NSO being ingested (e.g., they may know only a brand or street name of their compound), or that they are consuming an NSO at all, if mixed with marketed substances (e.g., cocaine, heroin), and the NPS/NSO drug market scenario is continuously changing. Furthermore, at least until recently many medical examiners did not test for NSOs, unless there was a specific reason to do so (16), with routinely used toxicology screens (i.e., immunoassays for heroin and its metabolites) not being able to detect NSOs (17).

Summarizing, it is still unclear whether NSOs and opioid analgesics play an important role in the development of opioid use disorder (OUD) outside the USA, and how much heroin users, either untreated or currently in opioid-assisted treatment, are attracted by NSOs. As long as NSOs are not routinely assessed/identified in drug screenings, their potential use could be an issue for example for prison inmates; for residents in therapeutic facilities; or in association with official driving license issues, if active opioid users want to submit apparently opioid-free biological specimens for testing, in order to retain or regain their license. Hence, the goal of the present study was to assess the prevalence and history of NSO use in an opioid (mostly heroin) addicted patient population, and its possible relationship with sociodemographic and clinical data.

## Methods

### Study Design

The present study analyzed data from a cross-sectional multicentre study focussing on the prevalence of multiple drug use in patients starting an in-patient detoxification from illicit drugs (18). Data from an additional participating center were added to the database. In the present analysis only patients with an opioid dependence as a primary diagnosis were included.

All ten participating institutions, located in the Western part of Germany (North Rhine—Westphalia), were members of an association of facilities and professionals in the field of drug treatment and drug detoxification (“Fachverband Qualifizierte stationäre Entzugsbehandlung Opiatabhängiger”). In the participating detoxification wards, patients routinely underwent at admission an anamnestic interview, which included questions concerning past and present drug use. A range of basic socio-demographic characteristics (e.g., age, gender, migrant background, current relationship status, living with children, current employment) were here recorded; for the purpose of the present study, the interview was standardized with regard to both questions and answers, and specific questions about NPS and NSO use were included. The laboratory analysis of the drug screenings routinely taken at treatment admission was expanded to include a range of NPS. This required an additional transfer of urine samples to an external laboratory and was not carried out by all participating facilities (see below). The study was reviewed and agreed upon by the ethics board of the Medical Faculty, University Duisburg Essen (File Number 18-8580-BO). Data were collected during the years 2018 and 2019.

### Recruitment, Inclusion and Exclusion Criteria

During the 8–12 weeks study period in a center, all of its eligible patients were invited to participate. If patients complied with this request, they were informed about study aims, study procedures, and data protection measures. In particular, patients were informed that their data were stored and analyzed in pseudonymous form; all recruited patients gave their written informed consent.

Exclusion criteria were as follows: insufficient understanding of the German language to fully understand the study information; presence of a cognitive impairment, including severe symptoms of intoxication or withdrawal, which prevented the full understanding of both study information and/or of interview questions (patients could be approached later during their treatment, if withdrawal or intoxication symptoms had receded); current occurrence of a severe psychiatric disorder (e.g., acute psychosis), which possibly impaired full understanding of the study information. Patients could object to study participation at any time, and without negative consequences. To be included, all patients had to present with a diagnosis of dependence from opioids/opioids, corroborated by an opioid-positive urine screening at admission.

### Assessments

The psychotropic drug use standardized questionnaire included the names of most widespread substances (e.g., heroin, methadone, buprenorphine, cocaine, cannabis, alcohol). One question was for “Other opioids (like Tramal etc.,)”; one other question was for “Illicit synthetic opioids (for example Carfentanyl, U-47700)”. For each substance or class of substances, patients indicated if they had ever used it at least once during their lifetime. If the patient reported a lifetime use of an “other opioid” or of a synthetic opioid, s/he was asked for its specific name. With regard to those substances with stated lifetime use, patients were asked on how many of the 30 days before treatment entry they had consumed them, with which typical daily dosage, and by which route of ingestion. Both questions and answers' formats were adapted from the European Addiction Severity Index (19, 20). After these questions about recent use, also details about previous use were asked for. This included levels of lifetime frequency (<5 times, 5–50 times, more than 50 times); historical years of regular consumption (defined here as at least weekly, e.g., every weekend); and years of daily/almost daily consumption. Comprehensiveness and feasibility of the interview were tested in a pilot study with 12 patients; questions and answering options were then improved where necessary. The interviews were carried out by medical staff members of the respective wards.

### Analytical Testing

In addition to the interview, urine specimens routinely sampled at admission from 6 out of 10 participating centers were sent to an external laboratory (LVR Klinik Viersen, head: Jürgen Sawazki). Four sites opted out of such additional urine analyses, to avoid additional workload. The determination of both NPS and traditional drugs of abuse (DOA) out of a urine matrix was performed utilizing Solide-Phase-Extraction (SPE), followed by a screening on a Gas Chromatography system coupled with a Time-of-Flight Mass spectrometer (GC-ToF-MS). Acetate buffer and beta-Glucuronidase/Aryl Sulfatase were added to 3 ml urine and incubated for 30 min at 56°C. Afterwards, DOA extraction was performed according to a validated methodology on a SPE cartridge (21). This step was followed by the injection of 1 μl of the extract into the GC-ToF-MS. To perform a sensitive analysis, all data were collected at a high detector voltage. A ToF system allows detecting every eluting analyte on a very high data rate, which allows the sensitive detection of very small amounts of psychoactive substances. This was felt here as particularly relevant, since most NPS are excreted out of the body in small concentrations and their metabolites in even smaller concentrations (22), with metabolites providing important information on when the drug was ingested and which drug was consumed. The separation of the mixture was performed in 14 min, and this was followed by an automated deconvolution, which allowed the determination of coeluting substances as well. Spectra of those drugs identified from the current analysis were then compared to largely available existing libraries' (e.g., SWGDRUG.ORG) spectra of already known drugs. These libraries are updated every 3 month. Modifications of the NPS occur on a regular basis. Changed substances will not be found in the existing libraries. After the changes, the mass spectrum of the chemical structure stays consistent, only the molecular mass changes. Looking at the similar mass spectrum, an experienced analyst can identify the class of the NPS, even if the specific substance is not found in the library. When suspicion of an NSO was arising, additional research was carried out to identify any further index molecule(s) [see (18)].

The Laboratory has identified several NSO in urine samples, for example U 47700, Methylfentanyl, Acrylfentanyl or Crotonylfentanyl. Additionally the detection of Mitragynin, the ingredient of Kratom, is carried out with the same method.

### Data Analysis

Interviews and printouts of the urine analyses' results were pseudonymized using a code based on letters from a patient's given name and his birthday. The documents were sent to the LVR Klinik Essen for data entry and statistical analyses. The statistical analyses were mostly descriptive; the comparison between NSO lifetime users and non-users with regard to age was carried out using independent samples *t*-test.

## Results

As previously outlined (18), 46% of patients admitted to the detoxification wards during the observation period were not included; about half of non-participants had not consented, and the remaining were not included because of either their medical conditions, which impaired the levels of collaboration, or language problems. Hence, *n* = 256 patients were here included in the study. Nearly two thirds of them were referred from opioid maintenance treatment (Table 1), either for detoxification from concomitant substance use, or for detoxification from the maintenance drug itself. High rates of other drugs use, apart from opioids/opioids, were here identified. In particular, those patients who were in an opioid maintenance treatment (*n* = 161, 62.8% of the total sample) at admission, frequently (68.9%) reported a current heroin use.

**Table 1:** Sample characteristics.

	**%**	***n***
**Age**
Min-max, median	20–62, 40	
Mean (SD)	39.8 (7.8)	
**Gender**
Male	77.7%	199
Female	22.3%	57
**Migrant background** ^*a*)^
yes	44.9%	115
no	55.1%	141
**Relationship status**
Permanent relationship	48.5%	124
No permanent relationship	51.5%	132
**Children in household**
yes	12.1%	31
no	87.9%	225
**Employment status**
Employed	12.9%	33
Unemployed	75.4%	193
Other	11.7%	30
**Daily smoker**
Yes	95.7%	242
No	4.3%	11
**Current additional substance use disorders**
Cocaine	30.9%	79
Cannabis	31.6%	81
Alcohol	37.9%	97
Benzodiazepines	28.1%	72
Amphetamine	7.8%	20
None	27.7%	71
**Current opioid maintenance**
Methadone, Levomethadone	52.7%	135
Buprenorphine	8.6%	22
Morphine	1.2%	3
Diamorphine	0.4%	1
None	37.1%	95

*Frequencies do not always add up to total sample size, due to missing responses*.

*^a)^Either the patient or at least one parent was foreign born*.

**Figure 1:**
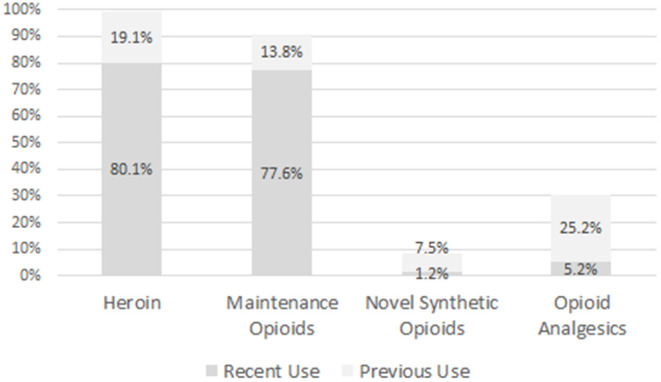
Proportion of patients reporting recent (last 30 days before admission to detoxification treatment) or previous use of several classes of opioids.

As can be seen from Figure 1, nearly all participants had used heroin during their lifetime, and some 80.1% reported its use over the 30 days prior to the current treatment initiation. More than 90% reported a lifetime use of maintenance opioids, and a lifetime use of opioid analgesics was reported by 30.4%; for 5.2% of them this was a recent use. In addition, 18 (26.8%) out of 67 lifetime users reported of daily use in the past. Of those 76 patients who reported a lifetime use of opioid analgesics, only 27 were able to mention the names of the molecules used, including 4 patients who named 2 substances. Named molecules included: tramadol (18 reports; 66.7% of the named substances), tilidine (5; 18.5%), morphine (5; 18.5%), codeine (2; 7.4%), and hydromorphone (1; 3.7%).

**Figure 2:**
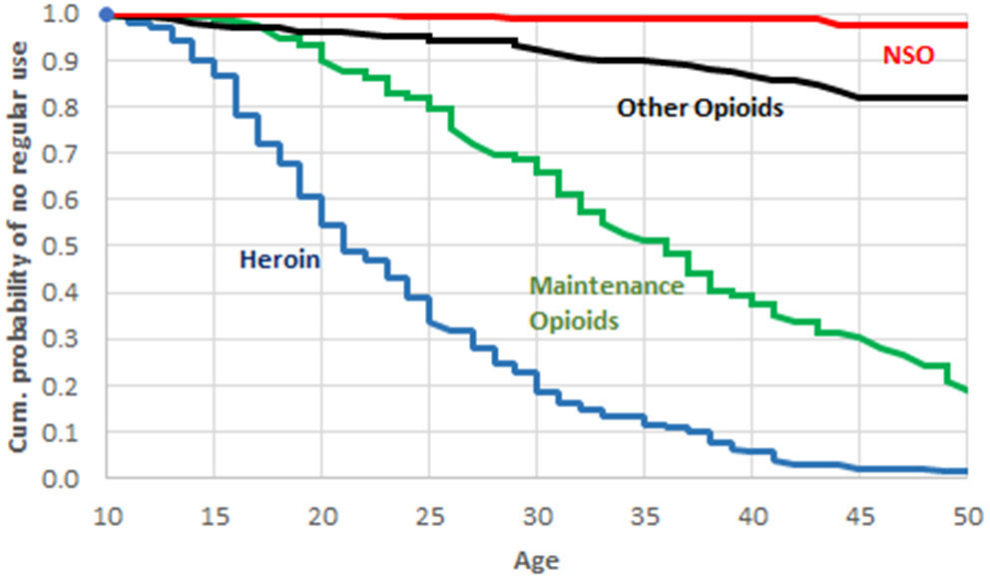
Cumulative probability of no regular use during lifetime.

Only *n* = 22 (8.7% of patients) reported an NSO use during their lifetime, and its recent use was limited to *n* = 2 (0.8%) cases; one of them reported use on 2 days, and the other one on 14 days, during the 30 days prior to admission. Of the 22 patients who reported a lifetime NSO use, 13 were able to name the respective substances. There were 10 reports of fentanyl, 2 of carfentanyl, and 1 patient reported of U-47200 and U-49200 use. Ten out of 22 NSO users had consumed them <5 times during lifetime; for 7 users, number of consumptions was between 5 and 50; and 3 users reported more than 50 consumptions. Two users did not indicate their NSO ingestion lifetime frequency. Only 4 patients (1.6% of the total sample) reported of a regular NSO use, e.g., at least weekly over several weeks, during their lifetime. As can be seen in Figure 2, for the study group as a whole there was a clear time sequence for the uptake of regular use of the 4 classes of opioids; more precisely, heroin was the first opioid/opioid, followed by maintenance drugs, then opioid analgesics, and finally NSO.

### Association of NSO Use With Age and Use of Other Drugs

Lifetime NSO users were about 4 years younger, on average, than nonusers (Table 2). While in the subgroups of younger patients (20–30-years-old) 6 out of 32 (18.7%) reported of a lifetime NSO use, in the groups aged 31–40 and 41–64 the respective rates were 8.3% (9 out of 108 patients) and 6.3% (7 of 111). Lifetime NSO users also showed statistically significantly higher rates of lifetime opioid analgesics use nad lifetime use of other Novel Synthetic Substances (such as herbal drugs, synthetic cannabinoids, synthetic stimulants), and a higher mean number of non-opioid substances consumed during lifetime.

**Table 2:** Opoid use history of patients with or without lifetime NSO use.

	**Lifetime NSO users (*n* = 22)**	**NSO non-users (*n* = 228)**	***p* ^*b*)^**
Age (mean, SD)	36.3 (7.8)	40.1 (7.7)	0.03
Lifetime opioid analgesics use^*a*)^	11 (45%)	60 (26.8%)	0.008
Lifetime number of non-opioid drugs (mean, SD)	10.5 (4.2)	6.5 (3.6)	<0.001
Lifetime use of other Novel Synthetic Substances	11 (50%)	61 (26.8%)	0.021
Current opioid maintenance treatment	11 (50%)	82 (36%)	0.19

*^a)^n = 20/n = 224 valid cases, respectively*.

*^b)^Chi^2^ or t-test, respectively*.

### Results From Urine Analyses

Six of the participating sites sent in urine specimens to a central laboratory. Thereby the urine samples of 181 out of 256 patients were analyzed using a broadband GC-ToF-/MS approach. Of these, 1 was positive for fentanyl; no fentanyl analogs were identified. Conversely, tilidine and tramadol were detected in 2 samples.

## Discussion

To the best of our knowledge, data assessing history of NSO use in opioid dependents starting a detoxification treatment, with verification of patient information with the means of enhanced laboratory measurements, were here first provided. Most typical, patients were males, older than 30 years, unemployed, with a history of both heroin use and opioid maintenance treatment. Nearly one third of patients were familiar with opioid analgesics such as morphine, codeine, tilidine or tramadol, but recent use was reported only by 1/20 of patients. About one in four of lifetime opioid analgesics users had used them daily, at least for some time. It seems therefore plausible to assume that at least ¾ or more of lifetime analgesics users had used this substance class without regular prescription (e.g., for a daily treatment of chronic pain). Lifetime use of NSOs was reported by 22 patients, including 2 patients who reported also recent (but still infrequent) use. According to the current laboratory results, no evidence was here identified supporting a possible heroin contamination with NSO.

Present data seem to be at odds with the “opioid crisis” described in a number of English speaking countries. Indeed, the time course of the use of different opioids during lifetime suggested here that heroin most typically anticipated the use of both opioid analgesics and NSOs. These findings are consistent with the relatively low rates of drug fatalities in Germany, where in 2020 some 1,540 deaths were recorded (23), including non-overdose deaths e.g., by suicide, or as a consequence of illicit drug use, e.g., from Aids or Hepatitis C transmitted by shared needles. This means a death rate of 1.9 per 100.000 population in Germany, compared with 21.3 (2019) or 27.7 (2020) in the USA, respectively, only including overdose deaths (24). NSO use was detected only in 5/1540 (0.3%) of German fatalities, and often in association with other drugs (23).

With clinical guidelines relating to the use of opioid analgesics being similar worldwide (25), in Germany the level of opioid analgesics prescription in non-cancer patients is comparatively low (26), although these patients are still presenting with levels of risk for developing a prescription opioid use disorder (27). However, no evidence of an opioid epidemic comparable to the US one has been described from Germany (28). These differences relating to non-medical prescription opioid use may be explained by a range of factors characterizing North America, including: higher level of overall prescription of psychotropics; a health-care system with smaller levels of regulatory access restrictions, hence possibly facilitating diversion; higher rates of patients' expectation of being provided with a potent/effective pain treatment; and strong opioid-related pharmaceutical advertising (29).

Only about 8.7% of patients reported here an NSO ingestion over their lifetime; patients reporting fentanyl use (*n* = 12) mostly regarded this drug as an NSO (*n* = 10/12), and not as an opioid analgesic. When a lifetime NSO use was reported, the typical frequency of consumption was irregular, and recent use was minimal; this was confirmed by urine screening results. Indeed, for none of the patients their NSO intake was the reason behind the admission to inpatient opioid detoxification treatment.

In 2017, European researchers alerted on the risk of illicit fentanyl mix used in place of heroin or contained in adulterated heroin itself (14). These and other warnings have led to an increased awareness of the risks associated with NSOs and to efforts to monitor such risks. Fentanyl and its analogues and/or metabolites were detected in in 23 out of 296 screened urine samples (7.8%) from opioid addicts receiving methadone maintenance treatment in Barcelona, Spain. No other NSOs were found (30). Fentanyl analogues were found in 6 out of 211 urine samples from patients admitted to emergency or addiction departments in the French Lorraine region. In 5 of these cases, its presence was justified by their therapeutic use as analgesics. Only patient abused fentanyl as recreational drug. No illegal synthetic opioid was detected (31).

A recent overview regarded opioid use in the European countries Germany, France, the United Kingdom, and the Netherlands. Using the same indicators as in the USA, it found no evidence of a current or emerging opioid crisis in these countries, with the exception of Scotland, where opioid-related harms showed a high rate (32).

In sum, in contrast to some countries outside Europe, fentanyl and fentanyl analogues do not appear, at present, to play a significant role in opioid use disorders across Europe, where only 0.5 (1) to 1.6% (33) of patients entering treatment for opioid use disorders mention fentanyl as a primary drug. A remarkable exception is Lithuania with two-digits rates of referrals who present with NSO problems. Similarly, as opposed to those recorded in the USA, rates of fentanyl-related fatal overdoses in Europe may be both lower (34, 35) and on the decrease as well (9). It must be noted, however, that due to their high potency NSO are usually used in very low amounts, and this may be associated with problematic detection issues in some laboratories. In addition to under-reporting, under-detection of cases of NSO use or of related fatal complications is possible (9).

The present study was conducted within the context of a broader study investigating NPS use in German patients undergoing in-patient detoxification treatment from illicit drugs, whose results were published elsewhere (18). There, self-reported lifetime consumption of NPS was frequent (32.6%) and concerned mostly synthetic cannabinoids, but regular use during lifetime and also recent use was rare. In contrast to that former publication, here the self-reported prevalence of NSO and opioid analgesics use was presented and discussed for opioid dependents only, the database was broadened by adding one more study site, the temporal pattern of regular use of different classes of opioids was included, details were given about which opioids had been used during lifetime.

## Limitations

One could wonder about those biases here introduced after having excluded from participation a large proportion (46%) of patients not willing or able to participate. In fact, it is unknown if these groups of patients could have presented with a radically different pattern of NSO use.

The majority (63%) of the sample were opioid maintenance patients, which roughly resembles the opioid dependent population in Germany [56% (36)]. It may be asked whether these patients are less likely than currently untreated opioid dependents to be taking drugs that include fentanyl. More than two thirds of the maintenance patients in detoxification treatment, though, were concomitant users of other opioids, and therefore at risk for current NSO use (which might possibly gain additional attraction by the fact that NSOs are not detected in routine urine analyses during opioid maintenance treatment). Regarding possible lifetime NSO and opioid analgesics use, it has to be taken into account that opioid dependence is a relapsing disorder, characterized by alternating periods of active use, opioid maintenance treatment (with or without concomitant drug use), and abstinence (37). There seems to be no strong reason to assume that those patients who were recently in maintenance treatment differ fundamentally from recently untreated opiate dependents, with regard to their lifetime substance use patterns.

Furthermore, this was a cross-sectional investigation, hence characterized by selection biases; for example, those opioid addicts who had died in the community after having ingested an NSO were here per definition not included. Previous NSO users were here significantly younger than non NSO users. This may suggest the presence of a cohort effect, hence one could argue that higher prevalence levels of NSO use will be possibly identified in OUD patients in the future.

Elaborated drug urine analysis (GC-ToF-MS) regarding NPS (including NSO) were carried out for 6 out of 10 study sites. In the remaining 4 sites drug urine screening was carried only using standard enzyme-immunoassays which do not detect NSO. We do not know whether detectable NSO use of patients might differ between sites with or without elaborated analysis. The 4 cities in which the non-participating sites were situated, ranked 2nd, 3rd, 9th, and 10th with regard to population size. If supply with “novel” psychotropic substances is associated with degree of urbanity, we do not expect a considerable influence of (non-)participation in the additional urine analyses on overall findings and conclusions. Self-reported recent use of NSO was *n* = 2 in the group of patients with elaborated urine analyses, and *n* = 1 in the other group.

Finally, although laboratory results may have helped to better interpret current findings, self-reports are unable to reveal the occurrence of an NPS/NSO use unknown to the consumer; this may have decreased the lifetime NSO use levels here reported. Users of fentanyl might be unaware of this, if it is mixed with heroin or other drugs, without the knowledge of the consumer (38). Given the near zero rate of urine-detected NSOs, this risk seems to be low in the studied sample. Also, it is possible that drugs sold in pill form are thought to be these drugs to the consumer, but can turn out to be fentanyl, but patients in the present sample did not report of using opiates sold in pill form. Moreover, users might be aware of having purchased an uncommon opioid, but do not know which class it actually belonged this to. Less than half of NSO users were able to report the name of the molecule which had been ingested; one could then argue that maybe some of these drugs were indeed opioid analgesics and not NSOs. So the present data might in part be more reflective of how many people are being told they are using fentanyl. How to assess lifetime use of such a kind remains a matter of discussion.

The present data might therefore in part be more reflective of how many people are actually being told they are using fentanyl. How to assess lifetime use of such a kind remains a matter of discussion.

## Conclusions

It is here suggested that both NSO and opioid analgesics may not play a relevant role in the development and the course of an OUD in German opioid dependent patients referred for an inpatient detoxification treatment.

## Data Availability Statement

The raw data supporting the conclusions of this article will be made available by the authors, without undue reservation.

## Ethics Statement

The studies involving human participants were reviewed and approved by Ethics Board of the Medical Faculty, University Duisburg-Essen. The patients/participants provided their written informed consent to participate in this study.

## Author Contributions

MS and NS designed the study, carried out the data analysis, and interpreted the results. MS wrote the manuscript. JS and LS designed and carried out analyses of biological samples and wrote a description of the technical details. TK, UB, SK, RS, TG, HE, AN, BZ, and KS discussed the study design, organized the acquisition of data, and contributed to the interpretation of results. FS reviewed earlier drafts of the manuscript. The authors agree to be accountable for all aspects of the work in ensuring that questions related to the accuracy or integrity of any part of the work are appropriately investigated and resolved.

## Funding

This study was conducted as part of project JUSTSO, funded by the European Union's Justice Programme—Drugs Policy Initiatives (Number: 806996—JUSTSO—JUST-2017-AG-DRUG). Urine analyses were financially supported by the Fachverband Qualifizierte stationäre Akutbehandlung Drogenabhängiger e.V.

## Conflict of Interest

The authors declare that the research was conducted in the absence of any commercial or financial relationships that could be construed as a potential conflict of interest.

## Publisher's Note

All claims expressed in this article are solely those of the authors and do not necessarily represent those of their affiliated organizations, or those of the publisher, the editors and the reviewers. Any product that may be evaluated in this article, or claim that may be made by its manufacturer, is not guaranteed or endorsed by the publisher.
